# Supplementary material for: Step-patterned survivorship curves: Mortality and loss of equilibrium responses to high temperature and food restriction in juvenile rainbow trout (Oncorhynchus mykiss)
Source: PLoS One. 2020 May 29;15(5):e0233699. doi: 10.1371/journal.pone.0233699 (PMC7259696; doi:10.1371/journal.pone.0233699)
Supplement: S1 Table — Standard deviation among individual fish are not available because fish were bulk weighed to minimize handling. The 10 individuals sampled from each of the treatment groups for the baseline group are included in these observed masses. (DOCX) [file pone.0233699.s001.docx]

**Step-patterned survivorship curves: mortality and loss of equilibrium responses to
high temperature and food restriction in juvenile rainbow trout (*Oncorhynchus mykiss*)**

Jennifer L. Gosselin and James J. Anderson

**Supporting** **Information**

**S1 Table. Mass of rainbow trout by bulk group of individuals, and average mass by bulk group, tank, treatment group and all rainbow trout tested.** Standard deviation among individual fish are not available because fish were bulk weighed to minimize handling. The 10 individuals sampled from each of the treatment groups for the baseline group are included in these observed masses.

| **Treatment Group** | **Tank #** | **Bulk group #** | **n** | **Bulk mass (g)** | **Average mass (g) of individual fish** | | | |
| --- | --- | --- | --- | --- | --- | --- | --- | --- |
|  |  |  |  |  | **Per bulked group** | **Per  tank** | **Per treatment group** | **All tested** |
| **Mortality** | 1 | 1 | 5 | 32.4 | 6.5 | 6.6 | 6.8 | 6.8 |
|  |  | 2 | 7 | 48.9 | 7.0 |  |  |  |
|  |  | 3 | 8 | 47.8 | 6.0 |  |  |  |
|  |  | 4 | 5 | 35.5 | 7.1 |  |  |  |
|  | 2 | 5 | 7 | 47.7 | 6.8 | 7.0 |  |  |
|  |  | 6 | 7 | 48.3 | 6.9 |  |  |  |
|  |  | 7 | 6 | 44.4 | 7.4 |  |  |  |
|  |  | 8 | 5 | 34.2 | 6.8 |  |  |  |
|  | 3 | 9 | 8 | 52.4 | 6.6 | 7.1 |  |  |
|  |  | 10 | 8 | 60.2 | 7.5 |  |  |  |
|  |  | 11 | 9 | 65.8 | 7.3 |  |  |  |
|  | 4 | 12 | 5 | 37.9 | 7.6 | 6.6 |  |  |
|  |  | 13 | 6 | 38.9 | 6.5 |  |  |  |
|  |  | 14 | 7 | 46 | 6.6 |  |  |  |
|  |  | 15 | 7 | 42.8 | 6.1 |  |  |  |
| **Loss of equilibrium** | 5 | 16 | 1 | 7.17 | 7.2 | 6.4 | 6.8 |  |
|  |  | 17 | 7 | 45.2 | 6.5 |  |  |  |
|  |  | 18 | 7 | 47.2 | 6.7 |  |  |  |
|  |  | 19 | 6 | 38.5 | 6.4 |  |  |  |
|  |  | 20 | 4 | 21.8 | 5.5 |  |  |  |
|  | 6 | 21 | 6 | 40 | 6.7 | 6.9 |  |  |
|  |  | 22 | 6 | 45.4 | 7.6 |  |  |  |
|  |  | 23 | 8 | 54.8 | 6.9 |  |  |  |
|  |  | 24 | 5 | 32.6 | 6.5 |  |  |  |
|  | 7 | 25 | 8 | 54.8 | 6.9 | 6.9 |  |  |
|  |  | 26 | 8 | 54.8 | 6.9 |  |  |  |
|  |  | 27 | 9 | 61.9 | 6.9 |  |  |  |
|  | 8 | 28 | 8 | 52.4 | 6.6 | 7.1 |  |  |
|  |  | 29 | 8 | 60.2 | 7.5 |  |  |  |
|  |  | 30 | 9 | 65.8 | 7.3 |  |  |  |
