# Supplementary figures and images for: Step-patterned survivorship curves: Mortality and loss of equilibrium responses to high temperature and food restriction in juvenile rainbow trout (Oncorhynchus mykiss)
Source: PLoS One. 2020 May 29;15(5):e0233699. doi: 10.1371/journal.pone.0233699 (PMC7259696; doi:10.1371/journal.pone.0233699)

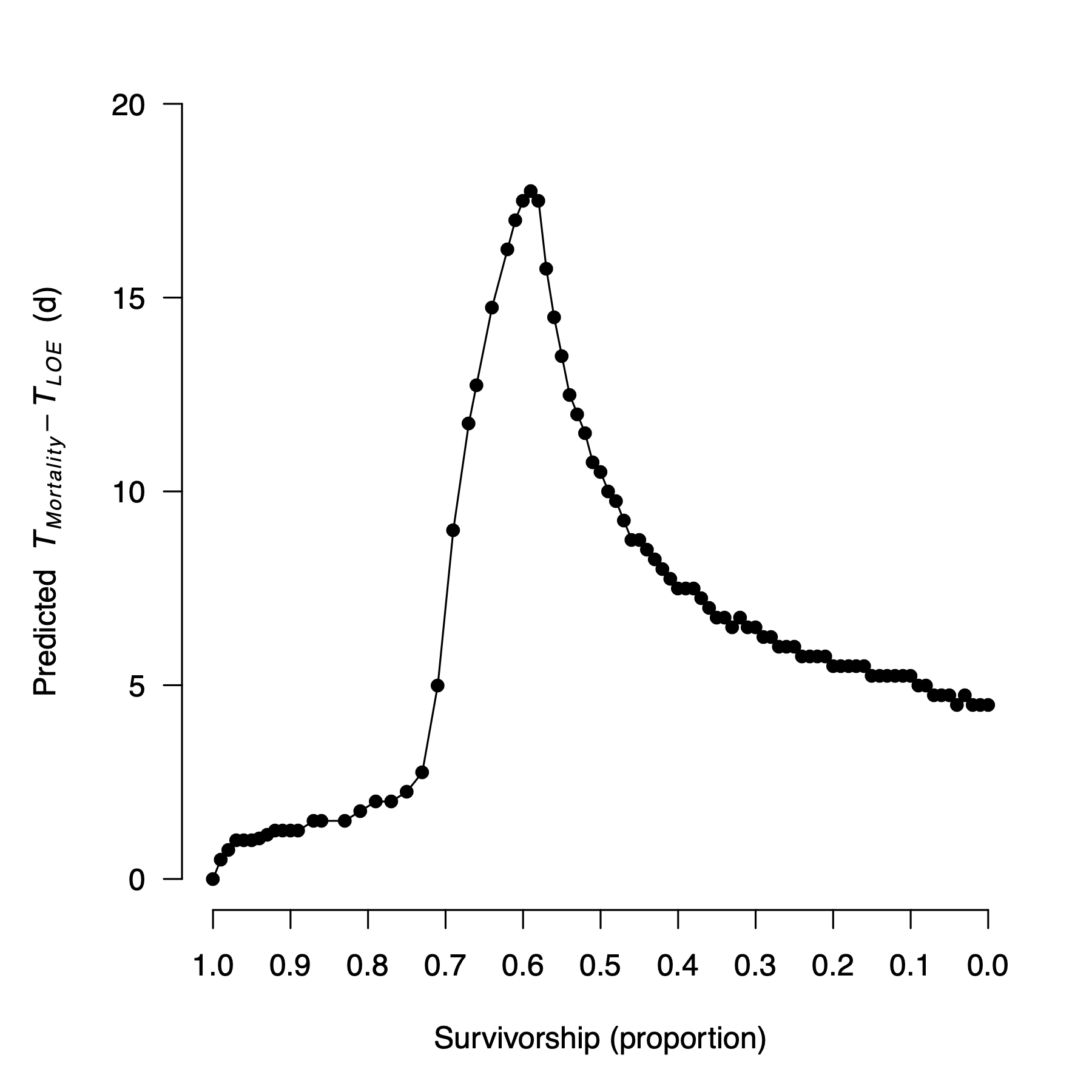

Supplement: S1 Fig — (TIFF) [file pone.0233699.s002.tiff]
