# Supplementary material for: Step-patterned survivorship curves: Mortality and loss of equilibrium responses to high temperature and food restriction in juvenile rainbow trout (Oncorhynchus mykiss)
Source: PLoS One. 2020 May 29;15(5):e0233699. doi: 10.1371/journal.pone.0233699 (PMC7259696; doi:10.1371/journal.pone.0233699)
Supplement: Attachment — Submitted filename: Response to reviewers.docx [file pone.0233699.s003.docx]

**I. Additional Editor Comments:**

***Editor comment #1:
Two reviews provide modest but substantial edits which would greatly enhance the manuscript.***

*Response:*
We greatly appreciate the review from you and from the two reviewers because the comments and questions have encouraged us to elaborate on some important concepts. These include putting our study in context of various studies about the upper critical temperature limits of rainbow trout, highlighting the relevance of our study to temperature tolerance across different systems, and further addressing the validity of loss of equilibrium as an endpoint in place of mortality in both ethical and ecological perspectives. We have edited our manuscript to address these major points and also minor comments as listed below. The line numbers referenced below for our edited manuscript are for the file with “Track Changes” turned on.

***Editor comment #2:
Particularly, Reviewer 1 suggests several recent publications of interest to this subject area and at least an acknowledgement of these other studies needs to be included within the revised text.***

*Response:*
We have added a paragraph to include references to the three publications the author suggested plus an additional one. Synthesizing and referencing these publications have helped to further justify our methods. So, we greatly appreciate this comment from Reviewer #1. For more details, see our response to Major Comment #2 of Reviewer #1.

***Editor comment #3:
As pointed out in the reviews, the manuscript is generally well written and the study is easy to follow. The authors should also place a bit more emphasis on the relevance of their findings since temperature tolerance studies are being viewed across many different disciplines.***

*Response:*
We have added to our ideas related to heat waves and increased frequency of extreme temperatures, across the life cycle of invertebrates and vertebrates, and referenced diverse taxa in lines 59-61, 294-296, 299-301, 425-426, and 431.

***Editor comment #4:
Reviewer 2 provides a valuable editorial point regarding the endpoint of the experiment. It is exceedingly important to justify this and why it made a difference in the data generated for this manuscript.***

*Response:*
The reason for looking into a surrogate endpoint in place of mortality was for animal ethics and welfare. Many studies collect data to determine lethal doses to 50% mortality, but in our study of multiple stressors on survivorship, we needed to observe mortality for all individuals. Thus, a surrogate endpoint of loss of equilibrium (LOE) served as a way to conduct our research in a more ethical manner.

The data generated by observing LOE did not necessarily make a difference in the overall conclusions, and that was what we were looking for in a surrogate endpoint.

In addition to animal ethics and welfare reasons, LOE is effectively a more relevant endpoint in the wild because individuals with compromised behavior are mores susceptible to predation. We have elaborated on these points and responded in more detail below, under the Major Comment by Reviewer #2.

**II. Journal requirements:**

**When submitting your revision, we need you to address these additional requirements.**

Our responses are in-line below.

***Journal requirement #1.*
Please ensure that your manuscript meets PLOS ONE's style requirements, including those for file naming. The PLOS ONE style templates can be found at**

<http://www.journals.plos.org/plosone/s/file?id=wjVg/PLOSOne_formatting_sample_main_body.pdf> and <http://www.journals.plos.org/plosone/s/file?id=ba62/PLOSOne_formatting_sample_title_authors_affiliations.pdf>

*Response:*
We have revised our manuscript to meet PLOS ONE’s style requirements, including updating the names of our files, the captions to our Figures and Tables captions, font size and style for headers.

***Journal requirement #2.* 
We note that you have stated that you will provide repository information for your data at acceptance. Should your manuscript be accepted for publication, we will hold it until you provide the relevant accession numbers or DOIs necessary to access your data. If you wish to make changes to your Data Availability statement, please describe these changes in your cover letter and we will update your Data Availability statement to reflect the information you provide.**

*Response*:
We have added our data to Dryad. <https://doi.org/10.5061/dryad.47d7wm38v> The data set will be published, contingent on acceptance of our manuscript for publication in your journal.

***Journal requirement #3.* 
Your ethics statement must appear in the Methods section of your manuscript. If your ethics statement is written in any section besides the Methods, please move it to the Methods section and delete it from any other section. Please also ensure that your ethics statement is included in your manuscript, as the ethics section of your online submission will not be published alongside your manuscript.**

*Response*:
We have completed the PLOS ONE Ethics statement Checklist (as submitted previously). We now have completed the ARRIVE NC3Rs Checklist, have provided additional information and/or clarification in lines 59-61, 119-134, 139, 141, 143-148, 150-152, 277-279, 394-397, and 410-411. Many of these edits are also related to reviewer questions and comments as listed below. We have written the ethics statement in the Methods section, and removed it from the Acknowledgments section.

***III. Reviewers' comments:***

***Reviewer's Responses to Questions***


***Question 1. Is the manuscript technically sound, and do the data support the conclusions?

The manuscript must describe a technically sound piece of scientific research with data that supports the conclusions. Experiments must have been conducted rigorously, with appropriate controls, replication, and sample sizes. The conclusions must be drawn appropriately based on the data presented.*** 

***Reviewer #1: No***

***Reviewer #2: Yes*** *Response to Reviewers #1 and #2:*
Given the mixed responses from reviewers, we interpreted that our manuscript was presented in a partially technically sound manner that needed further clarification and justification of our research experiments, results and conclusions.

With regards to controlled experiments with replication and large sample sizes, these are challenging to justify for complete survivorship curves with consideration of animal ethics and welfare. Through the approval process of our protocol by the University of Washington IACUC, we limited the number of animals tested as much as we could. We thus, in a sense, ran a replication in conjunction with a surrogate endpoint in place of mortality. The similar step-patterned survivorship curves in both treatment groups, in part, satisfy replication. Both groups showed mortality/LOE in an early period and a late period (Fig 1). For the energy density and percent dry mass data, we also made comparisons to the Baseline group and between treatment groups in Table 2 and in lines 273-277.

We understand that the lack of formal control and replicates are still a limitation of our study, even if our choice of fewer individuals tested were for animal ethics and welfare reasons. We have added lines 143-148.

Survivorship curves require many individuals for observation and analysis. In separate analyses with vitality modeling [1-3], we tried fitting the data from each of the four replicate tanks from the mortality treatment group for right-censored survivorship curves. These curves showed signs of step-patterns and similar fractions of individuals grouping into an early endpoint group (*f_1_*) and a late endpoint group (*f_2_*) (Table R1). But there were not enough individuals for robust statistical analysis for vitality modeling of survivorship curves. There were convergence issues with modeling and cautionary interpretation of these results is advised. Thus, we combined all individuals within a treatment group together for survivorship curve modeling (Fig R1, Table R2) [3]. For consistency, we also combined individuals within a treatment group together in our current study.

**Table R1. “Two population/process” model parameters for each of four tanks of system with water recirculating in parallel. See [2], for description of vitality model and parameters.**

**Fig R1. Survival curves fitted by the “one population/process” vitality model (red line, [2]) and the “two population/process” model (blue line, [3]) to the observed survival data (points) of juvenile rainbow trout exposed to increased water temperature and food restriction stressors.**

**Table R2. Parameters and AIC score of one- and two-population/process vitality models [1-3]. See [2], for description of vitality model and parameters. Blue font indicates parameters held constant in the model for both subgroups of fish. Highlighted rows represent best model by AIC selection, and models with ΔAIC<2.**

***Question 2. Has the statistical analysis been performed appropriately and rigorously? 

Reviewer #1: Yes***

*Response to Reviewer #1*:
Thank you.  ***Reviewer #2: I Don't Know***

*Response to Reviewer #2:*
Given the statement by Reviewer #2 about how they were “asked to review this paper looking specifically at the area of animal ethics and welfare”, we assume that there was less focus on the statistical aspect of their review. Nonetheless, we do want to take this opportunity to show that we have deliberated extensively on the appropriateness and rigorousness of our statistical analyses.

In earlier versions of our analysis, we applied more traditional analytical method of piecewise regression. This method is not appropriate because of the emphasis on the statistical method rather than actual physiological processes related to thresholds. We had thought about including these results in the Supplemental Materials, but then chose not to include them because they would complicate and detract from the results and discussions in the main paper. Here are the earlier text and results that we had thought about including in the Supplemental Materials:

**Methods R1. Piecewise regression of energetic reserves**

Mortality can occur when energetic reserves decline to a critical threshold [4]. Studies on fasting indicate that the rate of energy expenditure can be treated as a constant rate of loss [5]. By defining an energetic reserves index as *y*, its rate of depletion is *dy*/*dT* = *β* , where *β* is the constant rate of loss. Furthermore, by designating the critical energetic threshold as *y_crit_*, the time course of energy loss leading to starvation-induced mortality can be described by a piecewise linear regression as

$y_{T}=\left\{ \begin{aligned} y_{0}-\beta T, &0<T<t_{crit} \\ y_{crit}-\beta_{crit}T, &T>t_{crit} \end{aligned} \right.$	(Eq. R1)

where the energetic reserves index *y* is related to challenge time *T*, and *t_crit_* designates the switch from a *β* energy loss rate to a *β_crit_* energy loss rate at the critical energetic threshold y*_crit_*. By theory it follows that *β_crit_* should be zero since *y_crit_* is the starvation threshold and therefore mortality for *T* > *t_crit_* is assumed to be the result of starvation. Correspondingly, mortality occurring prior to *t_crit_* is assumed to be the result of heat stress.

The energetic reserves index *y* analyzed here is *M* percentage of dry mass by wet weight. LOE would also follow the form of Eq. R1 and be a suitable surrogate endpoint, if $t_{crit,LOE}<t_{crit,Mort}$; if $\beta_{LOE}=\beta_{Mort}$ and $M_{LOE,T}>M_{Mort,T}$ in the energetic loss period up to *t_crit_*; and $\beta_{crit,LOE}=\beta_{crit,Mort}=0$ and $M_{LOE.crit}>M_{Mort.crit}$ in the critical energetic threshold period.

We performed piecewise regressions using the function segmented from the R package segmented version 0.5-3.0 (R Core Team, 2018). In cases where *β_crit_* was not statistically different from 0 at α = 0.05, we simplified the regression to include only an intercept.

**Results R1. Piecewise linear regression of energetic reserves**

In a piecewise linear regression of energetic reserves, we estimated that an early phase of heat stress likely occurred between challenge days 0 and approximately 25 when *M* energy index decreased. After about 25 d of the challenge, the fish reached a *M_crit_* critical threshold of percent dry mass (Fig R2). The *t_crit_* times demarcating a switch from heat stress to starvation were equivalent between the Mortality and LOE groups (*t_crit,Mort_* = 23.8 d, 95% CI = 19.1–28.4 d; *t_crit,LOE_* = 27.2 d, 95% CI = 21.4–33.0 d). Together, these results suggest that fish dying or losing equilibrium in the early period experienced heat stress, while fish in the late period experienced energetic stress induced by critically low reserves.

However, the *t_crit_* times of about 25 d estimated in the piecewise linear regressions did not correspond to the increased instantaneous mortality rates observed from the survivorship curves at about 10–15 d (Fig 1). These results do not support the appropriateness of piecewise linear regressions of energetic reserves in representing early mortality caused by heat stress and late mortality caused by starvation.

**Fig R2. Percent dry mass of rainbow trout introduced to a challenge of heat and absence of food** for the (a) Mortality treatment group and (b) Loss of equilibrium (LOE) treatment group. Vertical line represents threshold *c* and shaded area represents its 95% confidence interval. Separate regressions between percent dry mass (*M*) and time (*T*) were tested for early (i.e., 0 < *T* < *t_crit_*) and late (i.e., *T* > *t_crit_*) periods of the challenge experiment. LOE group in this analysis does not include individuals that died before LOE occurred.

***Question 3. Have the authors made all data underlying the findings in their manuscript fully available?

The*** [***PLOS Data policy***](http://www.plosone.org/static/policies.action#sharing) ***requires authors to make all data underlying the findings described in their manuscript fully available without restriction, with rare exception (please refer to the Data Availability Statement in the manuscript PDF file). The data should be provided as part of the manuscript or its supporting information, or deposited to a public repository. For example, in addition to summary statistics, the data points behind means, medians and variance measures should be available. If there are restrictions on publicly sharing data—e.g. participant privacy or use of data from a third party—those must be specified.

Reviewer #1: Yes

Reviewer #2: Yes***

*Response to Reviewers #1 and #2:*
Our data are now on Dryad (<https://doi.org/10.5061/dryad.47d7wm38v>) and will be published, pending acceptance of our manuscript for publication in your journal.

***Question 4. Is the manuscript presented in an intelligible fashion and written in standard English?

PLOS ONE does not copyedit accepted manuscripts, so the language in submitted articles must be clear, correct, and unambiguous. Any typographical or grammatical errors should be corrected at revision, so please note any specific errors here.

Reviewer #1: Yes

Reviewer #2: Yes***

*Response to Reviewers #1 and #2:*
Thank you.

***Question 5. Review Comments to the Author

Please use the space provided to explain your answers to the questions above. You may also include additional comments for the author, including concerns about dual publication, research ethics, or publication ethics. (Please upload your review as an attachment if it exceeds 20,000 characters)***

Our responses to the points raised by reviewers are in-line below.

***Reviewer #1:***

***Major Comments:***

***Major Comment #1. This study investigated the survivorship of rainbow trout under heat stress and food restriction. By comparing mortality and LOE, the authors found a step-wised survivorship curve, with the acute response to be associated with heat stress and the chronic response to be associated with limited energy reserve. This manuscript is well present and easy to follow. However, the authors may want to provide some explanation to clarify some aspects.***

*Response*:
We added to our discussion more information that puts our study in context of existing literature on upper lethal temperatures for rainbow trout (see response to major comment #2) and a comparison between the timing of mortality and LOE (see response to major comment #3).

***Major Comment #2. It looks like the authors could provide more reviews of existing literature on studied topics. The upper lethal temperatures for rainbow trout are right around 24C, according to previous studies. (25.68C: Hokanson et al. 1977; 26.28C: Kaya 1978; Bear & MCMAHON 2007 and maybe more). That will provide background for readers about thermal requirement and tolerance of rainbow trout. Some of these studies also found acute mortality when acclimate fish at 24C, some fish were able to survive longer. Some of these papers also studied growth rate at high temperatures, which could be related to the chronic mortality due to limited energy reserves. Some thoughts on individual variability in the time of reaching the critical energetic threshold could be a nice addon.***
 *Response*:
Thank you. We greatly appreciate these comments and suggestions.

We have added a paragraph to lines 119-134 and lines 314-320.

***Major Comment #3. While LOE is ecological relevant, its reduced mobility of escaping from lethal condition often leads to mortality. Under thermal stress, LOE and mortality is very close, as seen in many critical thermal maximum studies. I wonder how mortality were controlled in the LOE tanks (see some of the minor comments). Other than that, I have some minor suggestions:***

*Response*:
Yes, we agree that LOE is ecologically relevant because it leads to mortality from predation. Under thermal stress, the timing of LOE and mortality can be close, but it can also be a difference of several days. In our study, the time between mortality and LOE, given a level of survivorship, was as great as 18 days (S1 Fig).

Also, as the reviewer noted, not all fish exhibited LOE. Because the responses of LOE and mortality likely occur from different but related processes, as we had reviewed in our discussion, we could not always control for mortality in the LOE tanks. Still, the majority of the fish we tested exhibited LOE.

More details on our responses to related review comments can be found under minor comment L74, L123, L125, L193-194, and the major comment by Reviewer #2.

***Minor comments:***

***L47, I am not sure why acute heat shock is a “non-energetic process”. Also, some fish examples will be more appropriate than C-elegans here.***

*Response*:
Yes, there is likely some energetic cost in maintaining homeostasis in response to heat shock. However, we were thinking that it is a negligible amount compared to the energetic loss from starvation. We have rephrased our ideas in lines 47-49. We have kept the references to C-elegans because these are studies showing different phenotypes related to step-survivorship curves, and we have also referenced a review paper on stresses in fish and heat shock.

***L74, Endpoints of LOE and mortality are often very close, especially when fish are under heat stress, e.g. critical thermal maximum. Did you expect to see a prolonged response of LOE here? In the later M&M section, you mentioned checking fish 3 times a day at 09:00, 16:00, and 22:00. LOE for several hours seem like a long time. How many fish from the LOE tanks died?***

*Response*:
Yes, we did expect to see a prolonged response of LOE before mortality occurred, particularly because we had chosen a temperature that would not immediately kill all fish, but that could result in starvation from a long-term stressor.

We have added a plot of the time difference between time to mortality and time to LOE (S1 Fig). This difference in time between endpoints was as great as 18 days. We added lines 277-278 and 394-397. Tracking times between LOE and Mortality for each fish within the Mortality treatment group was not possible because we did not individually tag the fish. Even if we had individually tagged the fish (e.g., with passive integrated transponder tags), we would have had to handle them to identify the fish at each observation. Another way to track fish individually, would be to house them individually. But we did not have sufficient facilities for the number of fish we tested.

Furthermore, we made observations at the very least three times a day at those times. We thought this would be sufficient given the duration of the entire experiment, which was about 60 days. Given the time the first author spent in the lab, observations were conducted more often than those three times per day throughout most of the experiment, as recorded in our data, and as plotted in our figures. The method we described in our manuscript was the minimum number of observations per day.

The number of fish in the LOE tanks that died without any signs of LOE was 26. This number can be calculated from the number of fish originally tested in the LOE group (N=90) as described in our Materials and Methods and from the sample size in Table 2 for the LOE group (n=64).

Additional phrases related to minor comments related to L123 and L125 below.

***L94, as the author mentioned “a heterogeneous population” before. I am curious how closely related are these hatchery fish, assuming there are some degrees of inbreeding.***

*Response*:
Unfortunately, we did not have the funds to conduct genetic analysis to observe and asses the degree of heterogeneity in our samples. We have contacted the hatchery to see if they could provide more information, but no further information was provided to us. We did note that we could not assess the degree of heterogeneity in our fish samples, and that we had to infer this from our survivorship and energetics data. We have edited lines 310-314.

***L97-100, these are probably unnecessary details, which are often seen in the AUP protocol.***

*Response*:
We did not have these sentences in our original submission to PLOS ONE. We added these details in response to reporting requirements and recommendations from the PLOS ONE Humane Endpoints Checklist and ethics statements needed in the Methods. Specifically, it was in response to “a scientific and ethical justification for the study design, including the reasons why humane endpoints could not be used, and discussion of alternatives that were considered but could not be used” and “all animal welfare considerations taken, including efforts to minimize suffering and distress, use of analgesics or anaesthetics, or special housing conditions”.

***L115 Some explanation of the choice of 24C. Some information/discussion of rainbow trout lethal temperatures will be useful (e.g. Bear et al., 2007). 24C seems like good choice as it is a lethal temperature for rainbow trout, but some fish could get acclimated and survive for a longer time.***

*Response*:
Yes, thank you very much. We have added a paragraph to provide detailed justification of our choice of 24°C. Please see lines 119-134.

***L123 Same comment as L74.***

*Response*:
Same response as comment related to L74. Added lines 150-152.

***L125 Same comment as L74.***

*Response*:
Same response as comment related to L74. We also added some text at lines 155-156 about different processes affecting LOE and mortality in the discussion.

***L135 parenthesis are often used as explanatory.***

*Response*:
Okay. Edits made in lines 165, 171, 172, and 173. Thank you.

***L191 I am not fully understanding the “the LOE group that experienced early mortality”.***

*Response*:
Rephrased in lines 222-224.

***L193-194 Since LOE occurrs before mortality events and they are related. Are you able to calculate the closeness of the timing between mortality and LOE?***

*Response*:
Yes. Based on the fitted curves, we determined the difference in timing and have added this plot (S1 Fig) to Supporting Information.

***L204, this is more like a statement of the result, rather than a hypothesis. It also occurred in other sections.***

*Response*:
We edited in lines 247-248, and also lines 165-169, 184-186, 326-327.

***L234 “Table 1” should be “Table 2”.***

*Response*:
Changed in line 273. Thank you.

***L238 Maybe the reason can be given for why “mean percent dry mass was higher for the LOE group than the mortality group”?***

*Response*:
Elaborated in lines 277-279.

***L326 Add year of reference***

*Response*:
Corrected in line 374. Thank you.

***L326 “spontaneous mortality” needs a definition***

*Response*:
“Spontaneous mortality” was a term used by IACUC at the University of Washington while we were developing the animal care protocol. We thought it was a common term. But since mortality and LOE are terms already defined and well used in our manuscript in sections prior to this line, we no longer think that it is necessary to qualify “mortality” further. We have deleted the term “spontaneous” from lines 390 and 401.

***L358-359 repetition of the statement made before (L355-L356)***

*Response*:
Deleted and rephrased to current lines 414-417. Thanks.

***Reviewer #2:***

***Major Comments:***

***Major Comment #1: I was asked to review this paper looking specifically at the area of animal ethics and welfare. While I have some strong concerns regarding choosing death from either starvation or heat shock as an endpoint, my concerns were at least partially mitigated by the portion of the study that was trying to validate the use loss of equilibrium as an earlier endpoint.***

*Response*:

Testing LOE to see whether it is a suitable surrogate endpoint in place of mortality was one of the goals of our paper. However, determining predictive estimates of time between time of mortality and time to LOE was beyond the scope of our paper.

The causes of LOE and mortality are numerous, and predictive estimates will depend on such mechanisms. We tried our best to synthesize relevant published literature on mechanisms of LOE and mortality and to consider our results in relation to these in our discussion (lines 325-381, 390-403).

Still, we have added a plot of the difference in these times relative to survivorship to the Supplemental Information to show that LOE generally does occur and to what extent it can occur before mortality (S1 Fig). We note that replicates would have helped to provide more robust estimates (lines 395-397), but we chose to test a limited number of individuals for reasons of animal ethics and welfare.

Given that our results showed a difference in time to mortality and LOE as great as 18 days at a given survivorship, LOE can be used as a surrogate endpoint with potentially a significant amount of time before mortality. LOE is thus an endpoint that can be incorporated into experiments in a more ethical manner than mortality, and it is ecologically relevant in context of predation. We note that further research is warranted and what processes could be involved in lines 397-403.

***Question 6. PLOS authors have the option to publish the peer review history of their article (***[***what does this mean?***](https://journals.plos.org/plosone/s/editorial-and-peer-review-process#loc-peer-review-history)***). If published, this will include your full peer review and any attached files.

If you choose “no”, your identity will remain anonymous but your review may still be made public.

Do you want your identity to be public for this peer review? For information about this choice, including consent withdrawal, please see our*** [***Privacy Policy***](https://www.plos.org/privacy-policy)***.

Reviewer #1: No

Reviewer #2: No***
 *Response:
Thank you for your review comments. We greatly appreciate them as they have strongly improved the quality of our manuscript. If we could, we would have thanked you both as anonymous reviewers in our Acknowledgments. But we understand and appreciate the journal’s reasonings related to equitable recognition and impartiality.*

***While revising your submission, please upload your figure files to the Preflight Analysis and Conversion Engine (PACE) digital diagnostic tool,*** [***https://pacev2.apexcovantage.com/***](https://pacev2.apexcovantage.com/)***. PACE helps ensure that figures meet PLOS requirements. To use PACE, you must first register as a user. Registration is free. Then, login and navigate to the UPLOAD tab, where you will find detailed instructions on how to use the tool. If you encounter any issues or have any questions when using PACE, please email us at*** [***figures@plos.org***](mailto:figures@plos.org)***. Please note that Supporting Information files do not need this step.***

*Response:*
We have uploaded our three figure files onto PACE. The files have been converted to valid tif files. After inspecting the uploaded files visually, the images are clear and have correct content, to the best of our knowledge.

*In compliance with data protection regulations, you may request that we remove your personal registration details at any time.*  [*(Remove my information/details)*](https://www.editorialmanager.com/pone/login.asp?a=r)*. Please contact the publication office if you have any questions.*

**References**

**1.** Anderson JJ. A vitality-based model relating stressors and environmental properties to organism survival. Ecological Monographs. 2000;70(3):445-70.

**2.** Li T, Anderson JJ. The vitality model: A way to understand population survival and demographic heterogeneity. Theoretical Population Biology. 2009;76(2):118-31.

**3.** Li T, Gosselin JL, Anderson JJ. Step-patterned survivorship curves represent competing stress mechanisms: superimposed survivorship curves defined by vitality theory. In progress.

**4.** Letcher BH, Rice JA, Crowder LB, Binkowski FP. Size-dependent effects of continuous and intermittent feeding on starvation time and mass loss in starving yellow perch larvae and juveniles. Transactions of the American Fisheries Society. 1996;125(1):14-26.

**5.** Caloin M. Modeling of lipid and protein depletion during total starvation. American Journal of Physiology-Endocrinology and Metabolism. 2004;287(4):E790-E8.
